# Novel Imaging Biomarkers for Brain PET Imaging

**DOI:** 10.3390/biom15040517

**Published:** 2025-04-01

**Authors:** Anne M. Landau

**Affiliations:** Translational Neuropsychiatry Unit, Department of Clinical Medicine, Aarhus University, 8200 Aarhus, Denmark; alandau@clin.au.dk

Positron emission tomography (PET) is a critical tool in the study of neurological diseases, providing non-invasive insights into the brain’s complex molecular and functional processes [1]. The Special Issue “Novel Imaging Biomarkers for Brain PET Imaging” in Biomolecules offers an overview of some of the latest advancements in PET imaging biomarkers, highlighting the possibilities and challenges of developing ligands for relevant targets while underscoring their significance in understanding neurological conditions.

The noradrenaline system has garnered significant attention for its role in mood disorders [2] and neurodegenerative diseases [3]. However, our understanding of its exact function and mechanism in disease pathogenesis and treatment has been limited, largely due to the absence of well-validated selective tracers to assess noradrenaline function and release in vivo in patients. Three original manuscripts in this Special Issue have employed the [11C]yohimbine tracer, which binds to α2-adrenergic receptors (α2-ARs) [4], offering insight into the noradrenaline system in vivo. 

Laurencin et al. used [11C]yohimbine PET to provide a detailed map of α2-AR binding in the brain of healthy human subjects, revealing the distribution of these receptors across various brain subregions and discussing their involvement in different neurological functions [5]. Importantly, they report specific sex-related differences in α2-AR binding and provide an interesting discussion on the topic. By identifying specific binding patterns of [11C]yohimbine, their study provides a deeper understanding of the receptor’s role in brain disorders. Moving to a disease context, Kemp et al. examined the correlation between quantitative electroencephalography (EEG) frequency patterns and α2-AR density in patients with Parkinson’s disease (PD) using [11C]yohimbine PET [6]. Their research suggests that EEG changes in PD patients reflect alterations in α2-AR density, which could serve as a non-invasive biomarker for tracking disease progression, including non-motor symptoms such as cognitive impairment. This finding highlights the potential of PET imaging with [11C]yohimbine in monitoring cognitive decline and personalizing treatment strategies, particularly in PD, where α2-ARs regulate neurotransmitter release, and their dysfunction may contribute to disease progression. The relationship between [11C]yohimbine binding and noradrenaline release was explored in a mechanistic study by Landau et al. [7]. They used a combined PET-microdialysis approach in a minipig model exposed to pharmacological challenges with different temporal effects and reported that reductions in [11C]yohimbine binding correlated with noradrenaline release, suggesting that [11C]yohimbine can be used as a surrogate marker of noradrenaline release [7]. This study reinforces the potential of [11C]yohimbine as a valuable tool for studying neurochemical dynamics in both psychiatric and neurodegenerative disorders. Taken together, these three contributions to the topic collectively underscore the potential [11C]yohimbine PET for in vivo monitoring of the noradrenaline system. 

Another active area of radioligand development research is that of tau tracers, which are relevant to Alzheimer’s disease (AD), other tauopathies, and research into brain injuries. Considering the importance of tau-targeting therapies for AD [8], PET imaging biomarkers of tau are of vital importance. The narrative review by Mohammadi et al. discusses the development and binding properties of tau PET tracers used for diagnosing AD [9]. They point out issues related to the different tracers, starting from the first successful tau tracer, [18F]FDDNP, which was initially designed for detecting amyloid β but also binds to tau aggregates, highlighting both amyloid and tau pathologies in AD. They then described more selective tau tracers, such as [18F]flortaucipir, which are now used clinically despite having off-target binding to targets like monoamine oxidase (MAO). Other tracers, like [18F]THK-5351 and [18F]MK-6240, also bind to additional targets such as neuromelanin, and their lipophilicity may contribute to white matter binding. To objectively assess effect size in PET studies, the authors report Cohen’s d for the contrast in tracer binding between healthy controls and AD patients with the goal of providing a rationale for selecting among the different structural classes of tau tracers available. The thorough review also notes limitations in the current literature, such as small sample sizes, reliance on clinical diagnosis rather than post-mortem confirmation, and potential disease heterogeneity, emphasizing the need for improved study design. 

A novel approach with potential in AD research is immuno-positron emission tomography (immunoPET), a non-invasive imaging technique that combines immunological targeting and PET to track radiolabeled monoclonal antibodies (mAbs) and related molecules [10]. Antibody imaging offers a precise and sensitive method for non-invasively analysing the cell surface phenotype in vivo, potentially providing more specific PET probes. The review by de Lucas et al. outlines the contributions of immunoPET to preclinical and clinical neurosciences [11]. They discuss that immunoPET in neuroimaging has been limited by the inability of intact antibodies to cross the blood-brain barrier (BBB) and mention strategies to enhance antibody and protein delivery to the brain, such as antibody engineering or modifying BBB permeability, including the use of focused ultrasound, which is being explored in preclinical studies. The authors highlight advances in immunoPET in neuro-oncology and neurological diseases, such as AD, where mechanisms such as neurotransmission, neuroinflammation and protein aggregation can be targeted. The review ends with an inspirational section on the perspectives for immunoPET, for example, in schizophrenia, where specific ligands are lacking and immunoPET can play a significant role in identifying biomarkers with subunit specificity, and in PD, where the search for a fully validated clinical alpha-synuclein PET tracer has been elusive.

The influence of obesity and metabolic factors on neurodegeneration has attracted increasing attention, with research proposing that chronic metabolic conditions can exacerbate the progression of neurodegenerative diseases. Obesity is associated with chronic low-grade inflammation and alterations in brain metabolism, both of which may contribute to the onset and progression of AD [12,13]. By visualizing the effects of obesity on brain function and metabolism, researchers can better understand how metabolic dysfunctions influence AD pathology. Müller et al. investigated the effects of diet-induced obesity in a long-term high-fat diet model in female mice on brain in vivo PET imaging with [18F]FDG and [18F]GE-180, tracers of glucose metabolism and inflammation, respectively [14]. Postmortem histological and biochemical analyses of specific neuroinflammatory markers were also performed. The main finding was that despite alterations in glucose metabolism and a significant increase in pro-inflammatory IL-1β mRNA expression, no significant changes in [18F]GE-180 uptake or histological evidence of glial activation or proliferation were observed. The authors suggest the lack of an effect on [18F]GE-180 may be due to differences in high-fat diet protocols compared to previous studies, or the use of only female subjects. Further studies are warranted to address the role of metabolic risk factors, such as obesity, in the prevention and management of neurodegenerative diseases.

A recurring theme is the role of sex in pathological mechanisms and the interpretation of neuroimaging data. Sex hormones like estrogen play a significant role in neuroprotection, cognition, and the prevention of neurodegenerative diseases [15]. Despite the widespread use of PET tracers for estrogen receptors in oncology, there are still significant gaps in developing effective PET tracers for the estrogen receptor family in the brain. The final contribution to this Special Issue is a perspective by Arjmand et al., which highlights the importance of estrogen signaling in the brain and discusses the challenges involved in developing PET tracers for identifying estrogen receptors within the brain [16]. This perspective emphasises the significance of exploring different subtypes of estrogen receptors in the brain, highlighting the distinct mechanisms of action of each. Unfortunately, there is a lack of specific, sensitive radiolabeled ligands for brain estrogen receptors and struggles in distinguishing between different subtypes, limiting our understanding of how estrogen affects the brain. Furthermore, hormonal fluctuations, particularly in women of reproductive age or those on hormone replacement therapy, can also complicate estrogen receptor imaging by altering receptor binding, making it difficult to distinguish between normal physiological changes and pathological alterations. The interpretation of PET data is further challenged by confounding factors like age, gender, genetics, and the presence of neurodegenerative diseases. As a result, there is still active research to establish reliable biomarkers for estrogen receptor activity to make PET imaging a more effective clinical tool. This perspective then presents several promising candidates based on computational simulations and emphasizes the gaps and areas that require further exploration to fully realize the potential of PET tracers for estrogen receptor imaging, particularly for the brain’s estrogen receptors. The ability to monitor estrogen receptor activity in vivo could open new avenues for therapeutic strategies, particularly for women at higher risk of neurodegeneration following menopause. For example, a recent study using labeled estradiol reported an increased binding ratio as a measure of estrogen receptor density during the transition toward menopause, irrespective of chronological age, and correlation with cognitive symptoms, which has high clinical relevance [17]. Targeting estrogen receptors with PET imaging can reveal important insights into how hormonal changes may influence the progression of diseases such as AD and PD.

In conclusion, the studies featured in this Special Issue present multiple approaches to PET brain imaging, reinforcing its growing potential in neuroscience in enhancing our understanding of brain health and disease. As technology advances and new biomarkers are identified for specific targets, PET imaging is poised to provide valuable insights into the pathophysiology of neurodegenerative diseases and psychiatric disorders. The ability to visualise key molecular processes in vivo offers a transformative opportunity to shape the future of clinical neuroscience, enabling earlier disease detection, more precise monitoring of progression, and more effective, individualised treatment approaches. 
